# Identifying distribution and accumulation patterns of floating marine debris in the Black Sea

**DOI:** 10.1016/j.marpolbul.2020.110964

**Published:** 2020-04

**Authors:** S. Miladinova, D. Macias, A. Stips, E. Garcia-Gorriz

**Affiliations:** Joint Research Centre, Via E. Fermi 2749, 21027 Ispra, VA, Italy

**Keywords:** Numerical modelling, Mesoscale circulation, Floating litter, Black Sea

## Abstract

The distribution and accumulation of floating marine debris in the Black Sea during the last few decades are analysed by the help of numerical modelling. An approach based on a mesoscale circulation model combined with a particle tracking model is applied. It is established that the litter distribution is nearly independent of the source location and is mainly controlled by the basin circulation system. The western gyre predominantly accumulates floating debris in summer. After the integration of the main cyclonic current in winter, the debris in the inner basin moves east. Retention zones along the south-western coast persist in time. The mean particle stranding time is estimated at about 200 days. Accumulation zones along the south-eastern and eastern coast are abundant in summer, and then move further northeast and north. Simulations demonstrate an increasing litter accumulation in summer on the North Western Shelf and shelf break.

## Highlights

• Floating litter is explored by eddy resolving and particle tracking models.
• The litter distribution is nearly independent of the source location.
• The western gyre accumulates floating debris in summer.
• Retention zones along the south-western coast persist in time.
• South-eastern accumulation zones move northeast and north in winter.

## Graphical abstract

**Figure:**
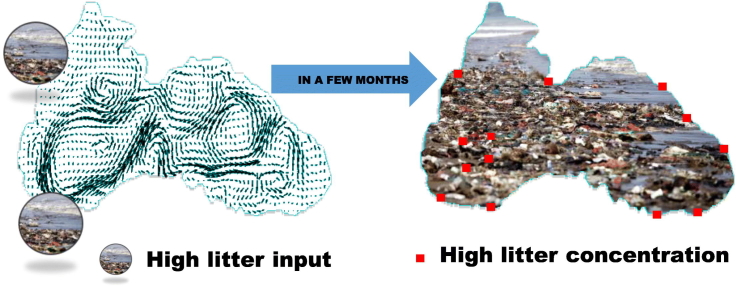

## 1 Introduction

The amount of marine debris in the marine environment has shown a steady increase in time (Ryan, 2015; Galgani et al., 2015; Barnes et al., 2009). Plastic typically constitutes the main part of marine litter with a proportion consistently varying between 60% and 80% of the total marine debris (Gregory and Ryan, 1997). Thus, globally, there is a rising concern about the risks and possible adverse effects of marine debris accumulation. It is obvious that litter enters the ocean from either marine or land based sources. Several statistical approaches have been developed in the last years to quantify the amount of plastics entering the oceans. They deal with visual counting from field data (Lechner et al., 2014; van der Wal et al., 2015; González-Fernández and Hanke, 2017), or statistical analysis at a global scale (Halpern et al., 2008; Jambeck et al., 2015; Lebreton et al., 2012). The methods vary in the size and properties of the litter taken into account. For example, visual counting is focusing on macro plastics (>2.5 cm), field measurements count from micro plastics to larger plastic items (Lechner et al., 2014; van der Wal et al., 2015), while statistics often disregard the litter size. Ship or aerial observations are focusing on buoyant litter and do not take into account the load under the observable surface water.

**Fig. 1:**
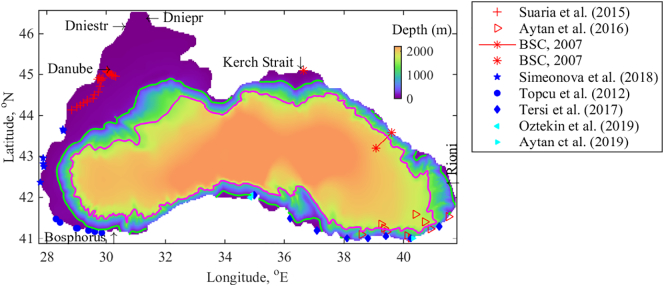
Bathymetry and location map of the Black Sea, main rivers and location of the survey sites. The 1500 m isobath is given in magenta and the 200 m isobath - in green. (For interpretation of the references to colour in this figure legend, the reader is referred to the web version of this article.)

Marine litter is considered as a crucial and complicated environmental problem in the Black Sea basin (BSC, 2007). The majority of the litter originates on land and river flow is the main source of litter into the basin (BSC, 2007). The litter input via the Danube into the Black Sea is estimated at 4.2 t per day (Lechner et al., 2014). In the Turkish Black Sea region, most of the municipal and industrial solid wastes, mixed with hospital and hazardous wastes, are dumped on the nearest lowlands and river valleys or into the sea (Berkun et al., 2005). Results of aerial surveys suggested that a significant amount of marine litter comes to the Russian Black Sea in late spring and early summer (BSC, 2007; Jeftic et al., 2009). Snowmelt and heavy rainfall during this period are thought to be key factors in carrying litter to the sea, as river discharge in this area is usually low at other times of the year. Vessel-based observations of floating plastic marine litter range from 6.6 to 65.7 items km^−2^ in the Ukrainian Black Sea and Kerch Strait. High litter densities peaking to 135.9 items km^−2^ (mean 30.9 ± 7.4 items km^−2^) were found by vessel-based survey in the north-western part of the Black Sea (Suaria et al., 2015). Locations of aerial surveys (BSC, 2007) and vessel-based survey by Suaria et al. (2015) are denoted in Fig. 1 with red symbols. Plastic is recognised as the dominant marine litter on the Black Sea beaches (Muresan et al., 2018; Simeonova and Chuturkova, 2019), in the sea surface (Suaria et al., 2015), and on the sea floor (Moncheva et al., 2016).

Floating litter items can be transported by the currents until they sink to the seafloor, be deposited on the shore or degrade over time (Andrady, 2015). It is expected that the quantities of litter will increase in the environment as a consequence of further direct introductions, however the likely paths and potential sinks or hot spots of accumulation are not clear. There is a lack of adequate data on the quantities, types and temporal trends of marine litter in the Black Sea. Available data are predominantly gathered on sandy beaches, while data for floating litter in the sea is exceptionally limited (Jeftic et al., 2009; Joint Black Sea Surveys 2017, 2018).

An assessment of beach litter shows that a large amount of debris has been observed in different areas of the southern Black Sea coast (Topçu et al., 2013; Terzi and Seyhan, 2017; Aytan et al., 2019; Oztekin et al., 2020). Locations of beach surveys are denoted in Fig. 1 with blue and cyan symbols. Identifiable foreign litter belonging to neighbouring Black Sea countries or another external source accounts for about 2.3% of total encountered litter (Oztekin et al., 2020). When the marine litter is classified according to the material types, plastic is the most prevalent type of litter in the Black Sea studies cited above. The south-eastern side of the Black Sea was found to be more polluted than its western side (Topçu et al., 2013; Terzi and Seyhan, 2017). Conversely, compared to the eastern side, the western side of the Turkish Black Sea is ~ 3.8 times more populated and hence its marine litter was expected to be denser. An extremely high litter density (1.51 ± 0.58 items m^−2^) is detected in Sarıkum Lagoon, which is located at about 42°N and 35°E (Oztekin et al., 2020). An even higher litter density with higher variance is estimated for Sarayköy Beach (2.10 ± 1.38 items m^−2^), which is located at about 41.02°N and 40.38°E (Aytan et al., 2019). Monitoring of marine litter along the Bulgarian Black Sea coast (Simeonova et al., 2017) shows that the beaches are highly polluted due to local sources, where cigarette butts and filters (OSPAR-code 64) are dominant. Since the highest marine litter accumulation is observed in summer, one can conclude that the accumulation is probably a result of recreational activities, increased tourist flow and wild camping. Therefore, the marine litter on the west coast of the Black Sea seems to be almost entirely of local origin. In summary, on the base of existing evidence the litter density on the west coast is less than on the south and east coasts.

Numerical modelling of floating marine debris (Martinez et al., 2009; Maximenko et al., 2012; Lebreton et al., 2012; Lebreton et al., 2018) have established and discussed the presence of accumulation zones in the main oceanic basins. On a European scale, regional seas have also been studied, such as the North Sea (Neumann et al., 2014), Mediterranean Sea (Mansui et al., 2015; Macias et al., 2019), East Mediterranean Sea (Politikos et al., 2017) and Black Sea (Stanev and Ricker, 2019). The modelling study by Stanev and Ricker (2019) has not identified any potential sites of floating litter accumulation in the inner basin during 2018. An estimation of litter spreading and recognition of preferred accumulation zones are essential in helping to raise the awareness of the marine pollution and, ultimately, to drive measures to reduce it. Numerical modelling techniques can help to establish the major drifting routes of marine litter. Our study proposes a methodology to track floating debris from source to their accumulation or beaching point and throughout the sea based on realistic descriptions of the Black Sea surface currents.

## 2 Materials and methods

### 2.1 Models

Our particle tracking approach involves a two-step procedure. First a 3D hydrodynamic model solves the equations of motion to describe water flow throughout the model domain. In the second step, virtual particles are introduced into the flow field and allowed to move freely through hydrodynamic forcing. For this study, six-hour currents are extracted from the 3D Black Sea hydrodynamic model (Miladinova et al., 2017). The hydrodynamic model comprises of the 3D GETM (http://www.getm.eu/) and 1D GOTM (https://gotm.net/), initialized on a high resolution 2 × 2 min latitude–longitude horizontal grid. The model bathymetry is produced from ETOPO1 global bathymetric grid with horizontal resolution of 1 min. The maximum depth of the model domain is 2200 m with a 70 levels general vertical grid, which is compressed toward the surface. The meteorological forcing from the European Centre for Medium Range Weather Forecast (ECMWF), available from http://www.ecmwf.int, based on 6-hourly records has been applied. It involves ERA-Interim project (1979–2018). Freshwater input has been estimated using the values from the Global Runoff Data Centre (GRDC, http://www.bafg.de/GRDC) runoff. The main Black Sea rivers, like Danube, Dniepr, Rioni, Dniestr, Sakarya, Coruhsuyu, Yesilirmak and Kizilirmak are considered herein. Recently, this model has been successfully applied to simulate the long term evolution of the Black Sea thermohaline structure and circulation (Miladinova et al., 2017, Miladinova et al., 2018).

Six-hour 3-D velocity fields extracted from GETM are then coupled to the Lagrangian particle tracking model Ichthyop v3.3 (Lett et al., 2008), which is used to track the floating litter in the sea. Ichthyop is a free Java tool designed to study the effects of physical and biological factors on the ichthyoplankton dynamics. Ichthyop movement sub-model simulates the horizontal advection, vertical advection, horizontal dispersion, vertical dispersion, and particle buoyancy. Since wind driven currents are already taken into account in the GETM hydrodynamic data, no additional wind stress is applied to the motion of particles. The same numerical approach has been successfully applied to study the circulation and accumulation of floating litter in the Mediterranean Sea (Macias et al., 2019). We assume that debris particles have density 0.9 g cm^−3^ that allows them to float in the water. The direct effect of the wind drag on the individual particles is not considered, since small floating plastic fragments are supposed to move under the sea surface in agreement with previous modelling approaches (Macias et al., 2019). Our model do not consider Stokes drift, wind drag, particle biofouling and settling. It is established that surface currents play major role in marine debris accumulation (Kubota, 1994; Martinez et al., 2009). Kubota (1994) found that Stokes drift does not significantly contribute to debris transport, while it can be important for kelp and oil distribution (Fraser et al., 2018; Drivdal et al., 2014). Our simulations are focused on small plastic items or on those large plastic items which remain mainly below the sea surface. For slightly buoyant (e.g., polyethylene, polypropylene) particles, the existence of biofouling leads to an increase of the mean particle density and its sinking. In this way the microplastic particles would be largely below the surface and thus less affected by the wind drag and Stokes drift (Chubarenko et al., 2016). According to the analysis of Pereiro et al. (2018) and Yoon et al. (2010), the wind drag for particles of a density comparable to that in our simulations should always be smaller than 1%. So, our approach might only introduce great bias in the presence of extreme events.

The release site of each particle is selected at random in the most likely debris generation areas specified in the following subsection. Ichthyop can represent one of the two different particle behaviours at coastline: bouncing or beaching. Ichthyop cannot represent both particle behaviours simultaneously. When bouncing is considered, the particle will return along the same trajectory it arrived on, but travelling in the opposite direction. Stranding of particles is not included in bouncing scenario, so when particles are found on shoreline grid boxes, they are bounced back to the sea, still considered to be part of the computational process. In the case of beaching - when a particle reaches the shore, Ichthyop stops any further displacements. From that moment onward the particle does not interact with its environment and remains trapped on the beach. The accumulation of beached particles is accounted for by the model until the end of the simulation period. We choose these two particle behaviours to calculate separately the litter accumulation zones when the litter does not accumulate on the beach (bouncing behaviour) and when the litter particles that reach the beach do not return back to the sea (beaching behaviour).

### 2.2 Inputs of marine litter

**Fig. 2:**
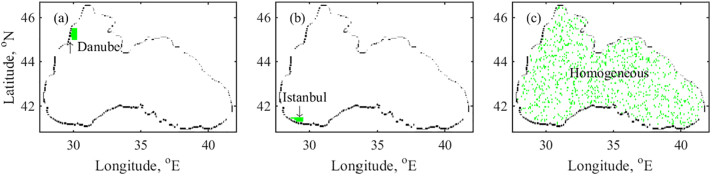
A graphical representation of the three particle release cases. (a) Danube – watershed area; (b) Istanbul – coastal population density and (c) homogeneous.

Accurate data for the total amount of litter input to the Black Sea is not available, however it is established that litter enters the Black Sea from either marine or land based sources. Faris and Hart (1994) estimate that 80% of the marine litter enters the ocean by land. Debris of terrestrial origin reaches the Black Sea mainly through runoff; via storm drains and waterways accessing areas where garbage is not adequately controlled (Jeftic et al., 2009). To tackle the inputs of marine litter, we use a scaled approach to define the particle release locations. Our input scenarios are based on Halpern et al. (2008), using three of their data layers - watershed area, coastal population density and shipping, in order to define the input of particles to the model. The main watershed based input is estimated to be at the Danube river mouth (Lechner et al., 2014; Suaria et al., 2015) and that based on the population density – is the Black Sea region of Istanbul (Berkun et al., 2005). The amount of marine based litter is assumed to be much smaller than the land based litter (Halpern et al., 2008). Fig. 2 indicates the position and size of the selected release (debris generating) zones, namely, the Danube and the Istanbul zone. A homogeneous release is also chosen as the initial distribution for the particles. This choice is determined by the lack of data set describing the litter distribution in the Black Sea.

### 2.3 Simulations

Depending on the particle release zones and particle coastal behaviour (bouncing or beaching), we consider 6 different scenarios (Table 1). The choice of the advection time is an important factor for the identification of marine litter retention or accumulation (Martinez et al., 2009; Neumann et al., 2014). In the Black Sea, advection time from a month to a year seems to be a good approach given the high speed of the currents (Miladinova et al., 2017). Longer simulations provide little knowledge because of the very high coastal retention rate. For this reason, the onset of particle release occurs in the beginning of the year, when the initial particle concentration in the basin is set to zero. Next, in the beginning of each month 1000 particles are released from a selected debris generating area (Danube, Istanbul, or homogeneous scenario). Then the particle distribution and accumulation through the basin by the end of the year are recorded and analysed. A monthly particle release is applied to account for a possible temporal/seasonal effect on particle distribution. The total amount of particles released is 12,000 per year. This amount represents an overall mean density of 0.028 particle km^−2^ or 0.325 particles per model grid box. Since the particle concentration in the basin is reset to zero in the beginning of the each year, the particle distribution during the year does not depend on the memory of previous years. To identify recurring debris locations during the most recent two decades, annual particle tracking simulations are performed from 1999 to 2018. Then, the mean number of particles in each grid box for the 20-year period is calculated. For trend analysis, the number of annual simulations has been increased to 40 (1979–2018). Based on 40-year runs, we try to detect potential statistically significant trend in particle concentration. We repeated the annual simulations for multiple years only in case of scenario D1 and D2 (Table 1).

**Table 1:** Scenario setup.

Scenario name	Release zone	Coastal behaviour	Annual simulation
D1	Danube	Bouncing	1979–2018
I1	Istanbul	Bouncing	2018
H1	Homogeneous	Bouncing	2018
D2	Danube	Beaching	1999–2018
I2	Istanbul	Beaching	2018
H2	Homogeneous	Beaching	2018

In order to study particle accumulations that are independent of the number of released particles, a relative particle density (RPD) percentage is introduced as RPD (%) = (number of particles in a grid box/total amount of released particles) x 100. For increased clarity when discussing the surface particle accumulation through the year, RPD values are grouped into a small number of bins. The bins are chosen based on the extensive analysis of RPD values, so the bin ranges cover gradually increasing RPD and each bin contains RPD values. We define accumulation zones in the Black Sea by using percentage thresholds of 0.02% (RPD ≥ 0.02%). This means that if 12,000 particles are released, grid boxes prone to litter accumulation have equal to or >2.4 particles per grid box. Therefore, these grid boxes contain at least 7.4 times more particles than in a homogeneous particle distribution (0.325 particles per grid box). The upper RPD boundary denotes zones with particularly high particle concentration. Five bins within the intervals [0.02; 0.04), [0.04; 0.06), [0.06; 0.1), [0.1; 0.15) and ≥0.15% are defined in the case of particle bouncing. Six bins within intervals [0.02; 0.1), [0.1; 0.2), [0.2; 0.5), [0.5; 1), [1; 2) and ≥2% are chosen in the case of particle beaching.

## 3 Description of the circulation

**Fig. 3:**
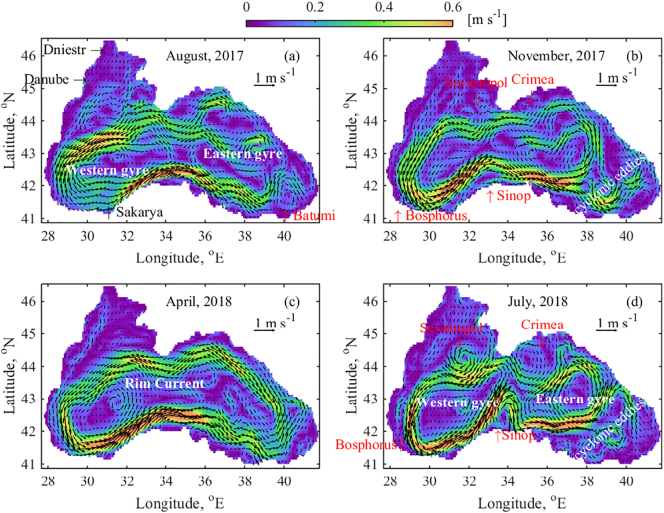
The Black Sea surface circulation based on monthly mean velocity vectors averaged over upper 10 m (a) August 2017; (b) November 2017; (c) April 2018 and (d) July 2018. The colour bar represents the surface speed [m s^−1^], while arrows show both speed and direction. Locations of Danube, Dniestr and Sakarya rivers, as well as Sinop, Sevastopol, Bosphorus and Crimea anticyclonic eddies are indicated. (For interpretation of the references to colour in this figure legend, the reader is referred to the web version of this article.)

Before discussing the results of the particle tracking scenario simulations, we shortly describe the circulation dynamics in the basin. The general circulation of the Black Sea is driven by the wind stress as well as by the large freshwater input from the Danube River and other big rivers. The circulation is further constrained by the steep topography around the basin periphery that consists of narrow shelves and a maximum depth of around 2200 m. There exists a major basin wide cyclonic circulation (Rim Current) with well pronounced Western and Eastern gyres (Oguz et al., 1992). The Black Sea circulation system involves a spatially complex structure, dominated by energetic mesoscale features (Korotaev et al., 2003; Kubryakov and Stanichny, 2015; Miladinova et al., 2017). The mesoscale circulation is characterised by a class of energetic phenomena of spatial scales ranging from about ten to several hundred kilometres and time scales ranging from a few days to several months. The spatial and temporal resolution of the GETM/GOTM configuration enables the simulation of such events, in particular the Black Sea eddies that may be responsible for debris spreading and accumulation. Further, we examine how surface circulation affects the marine litter accumulation and explore the possible parallels between the strength of the Rim Current and the variation of mesoscale eddies, and retention zones at annual or multi-annual scale. A detailed representation of the surface circulation is considered in four different cases (Fig. 3). The choice of time periods to explain surface circulation is closely related to several specific RPD distribution time periods presented in 4, 5.

In Fig. 3, examples of the velocity fields in 2017 and 2018 are given in order to highlight several circulation features that have major effect on the distribution and accumulation of marine litter. The colour bar is fixed in the interval 0–0.6 m s^−1^ for all figures in order to make more apparent the comparisons of the current speed. One general Black Sea feature is the strengthening of the basin scale cyclonic circulation in winter and the intensification of anticyclone activity in the warm period. Typically in summer, many anticyclonic eddies are formed between the shelf edge and the Rim Current. However in August 2017, the Rim Current is only partially separated into two gyres (Western and Eastern) and anticyclone activity is not yet well expressed. The emergence of Batumi anticyclone eddy is well visible (Fig. 3a). The Western gyre is integrated and encompasses the entire inner western basin. The Eastern gyre is weaker and resides in the eastern basin, along with several energetic cyclonic eddies. These eddies are spread in the east and northeast of the basin and their centres move in a counter-clockwise direction. The eastern pathway of the Eastern gyre meanders in relation with the surrounding eddies. The Rim Current has the highest speed along the Anatolian coast (32–35°E) and along the North Western Shelf (NWS). Surface circulation on the NWS is intense. In November 2017 (Fig. 3b), the process of two gyre system integration is visible. In particular, the Western and Eastern gyres merge and the energetic cyclonic eddies from the north and north-eastern part join the integrated system. However, several cyclonic eddies in the easternmost part of the basin continue to move independently. The Rim Current pathway is shaped by several anticyclonic eddies (Sevastopol, Crimea, Bosphorus and Sinop). Surface circulation on the NWS is weak and the south-western flow of the NWS rivers is clearly visible.

The Rim Current is integrated and strengthened in April 2018 (Fig. 3c) with a mean speed that exceeds 0.5 m s^−1^. The current is extremely fast (>0.6 m s^−1^) along the western and central part of Anatolian coast. The Western gyre tends to form by splitting the Rim Current at about 32°E into two branches. The surface activity on the NWS is dominated by the anticyclones in the vicinity of Danube and Dniestr. Further in July 2018 (Fig. 3d), the separation of the Rim Current into two gyres is nearly completed and the formation of strong anticyclonic structures is already visible. Both gyres exhibit very high speed and their pathways are shaped by several energetic anticyclonic eddies (e.g. Sinop, Sevastopol, Bosphorus and Crimea). The easternmost part of the basin is occupied by vigorous mesoscale cyclonic eddies. The cyclonic (anticyclonic) eddies are usually characterised by upwelling (downwelling) within the eddy, which lifts (pushes down) and vertically displaces materials inside the eddy. In this way, coastal anticyclonic eddies transport land based materials offshore and prevent the marine based materials to be deposited on the coast. A counter effect is that mesoscale cyclonic eddies support debris formation. The surface motion on the NWS is amplified in July 2018 and the freshwater plume from the NWS rivers first turns north, then east, and then flows south to the Western gyre. The back flow from the Bosphorus eddy along the western coast toward the NWS is clearly visible in Fig. 3d.

## 4 Model validation

**Fig. 4:**
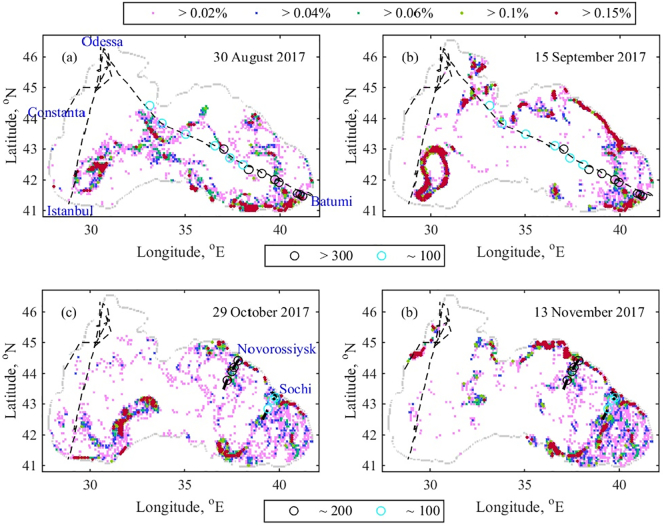
RPD (%) bins are presented by different colours, while EMBLAS-II data with density ≥ 100 items km^−2^ acquired by surveys is presented by circles. All survey transects are marked by the black dash line. (a) on August 30th; (b) on September 15th; (c) on October 29th and (d) on November 13th. (For interpretation of the references to colour in this figure legend, the reader is referred to the web version of this article.)

The study focuses on the spreading and accumulation of marine litter. We check the model ability to represent the real physical system. The ship or aerial based surveys are crucial to assess local patterns over a short period of time, however the data coverage remains sparse in the Black Sea. Recently, under the Project “EU-UNDP Project ‘Improving Environmental Monitoring in the Black Sea’ (EMBLAS-II)” (http://emblasproject.org/), monitoring of floating marine litter (>2.5 cm) is performed based on visual ship observations. Observation positions, transects and acquired data for 2017 can be found in the Scientific Report – Joint Black Sea Surveys 2017 (2018). The monitoring activities cover short time intervals and have limited spatial coverage. We plot in Fig. 4 the main monitoring transects of EMBLAS-II and places where marine debris has been identified (with density about or >100 items km^−2^). Four main transects/surveys are noteworthy – (i) Constanta-Odessa- Batumi-Constanta (operated by R/V Mare Nigrum from August 26 – September 07, 2017); (ii) Odessa-Istanbul-Constanta-Batumi (operated by ferries through 2017); (iii) Gelendzhik-sea centre-Novorossiysk (R/V Borey, during 24–28 October 2017); and (iv) Sochi-Adler region (R/V Katran, during 14–16 November 2017).

In order to evaluate our model against the data collected during these surveys, the bouncing particle model is run in the beginning of 2017. The first particle release occurs on January 1st, when 1000 particles are released from the Danube source (Fig. 2a) and then the same release is repeated in the beginning of each month. The Danube source is selected due to the fact that it is the most anticipated source of marine litter (Halpern et al., 2008). The first evaluation of RPD is done on August 30th and the second on September 15th (Fig. 4a and b). These dates are close to dates of survey (i). The next RPD estimations are done on October 29th and November 13th (Fig. 4c and d) in order to be close to the survey dates (iii) and (iv).

There are several similarities between our results in Fig. 4a, and data of survey (i). In particular the simulations suggest a high concentration of particles accumulating in the inner basin on August 30th. The transect of survey (i) in the eastern basin crosses zones with RPD ≥ 0.05%, while the transect of survey (ii) extends laterally from the area with RPD ≥ 0.05%. Since the timing of survey (ii) is not available, the accumulation zones in the western basin in August 2017 might be not recorded by the survey (ii). Our results also closely match the data from surveys (iii) and (iv), particularly on October 29th for survey (iii). When our predictions differ from the survey data, we anticipate this to be mostly due to the intense cyclonic/anticyclonic circulation in the studied area. Strong surface circulation causes rapid changes in the particle distribution over short periods of time, which could not be identified by a few records. For example, on September 15th (after the survey (i) period) the particle accumulation zones in the eastern basin are destroyed, while even stronger accumulation zone is formed in the western basin (Fig. 4b). The existence of accumulation zones in the centre of the western basin is related to the strong cyclonic circulation in the area (Fig. 3a). EMBLAS-II concludes that the floating litter in the eastern basin is about 138.6 items km^−2^, which much more than the litter in the western basin (23 items km^−2^). Based on RPD values along the survey transects presented in Fig. 4, one could draw a similar conclusion. However, our results show zones with high RPD which have not been crossed by the survey vessels. The EMBLAS-II concluding remarks clearly state that the monitoring organization, protocol implementation and reporting still need to be improved, together with the improvement of the spatial and time coverage.

## 5 Results and discussion

### 5.1 Scenario analysis

**Fig. 5:**
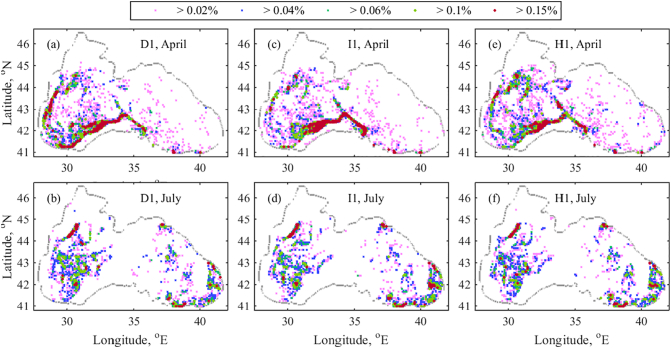
RPD (%) for 2018 in the case of bouncing particle behaviour near the coast. (a) D1 at the end of April and (b) – end of July; (c) and (d) - I1; (e) and (f) – H1. RPD (%) bins are presented by different colours. (For interpretation of the references to colour in this figure legend, the reader is referred to the web version of this article.)

Six different scenarios are run in the beginning of 2018 (Table 1), studying two forms of coast litter interaction and three sources of particle release. For evaluating a possible seasonal effect on the particle distribution from the release zones, two snapshots of each scenario (at the end of April and July) are shown in Figs. 5 (bouncing) and 6 (beaching). Interestingly, at the end of April the particles tend to accumulate along the Rim Current in the Western gyre and especially in the Rim Current south central part for all 3 bouncing scenarios (upper panel in Fig. 5). We found this result insightful, since the particle accumulation zones are almost entirely independent of the location of the release source. Thus, the circulation patterns govern the pathway of floating litter. The main difference between RPDs for the three release zones, shown in Fig. 5, consists of lower RPD in the south-western part of the Rim Current for the Istanbul scenario. The largest concentration of marine debris in April 2018 is likely to accumulate in the inner part of the Rim Current south pathway, where Rim Current speed is the highest (Fig. 3c). A large concentration of particles is also expected along the Rim Current in the northwest shelf break. In the case of Danube scenario, debris with RPD >0.15% can be seen along the south-western path of the NWS river plume. Since the Western gyre has begun to form in April 2018, the majority of the particles are locked inside the Western gyre. The Eastern gyre collects slightly more particles when homogeneous release is applied.

**Fig. 6:**
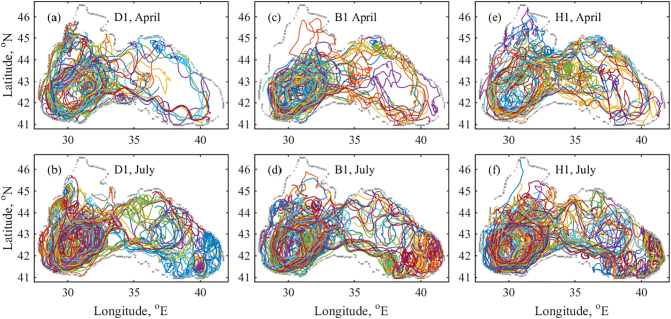
Particle trajectories for 2018 in the case of bouncing particle behaviour near the coast. (a) D1 from the begging of January to the end of April and (b) from the begging of May to the end of July; (c) and (d) - I1; (e) and (f) – H1. Each 200th trajectory is represented in random colour. (For interpretation of the references to colour in this figure legend, the reader is referred to the web version of this article.)

The particle distribution changes considerably in July 2018. The formation of a two gyre system (Fig. 3d) results in two distinct particle accumulation regions – eastern and western region. While the western accumulation region is compact and inside the Western gyre, the eastern region consists of several separate retention areas. Debris with high litter abundance is suggested to retain along the easternmost shelf due to several energetic cyclonic eddies in the region (Fig. 3d). Less concentrated debris is expected to arise in the Eastern gyre centre. It is worth noting the presence of a small patch with a high RPD north of Novorossiysk. In general, the debris distribution in July differs considerably from that in April. Lower accumulations can be found along the south path of the Rim Current, while larger accumulations are likely to form in the eastern basin, and especially along the south-eastern and eastern coast. RPD on the NWS between 44 and 45°N tends to increase in July due to typical high Danube discharge in May–July and the subsequent strong anticyclonic activity. It is worth mentioning that in July the similarity between the three RPD patterns (Fig. 5b, d and f) is even more striking.

Simulated trajectories of every 200th particle from the begging of January to the end of April 2018 are shown in the upper panel of Fig. 6 (20 in total per each sub-plot). Similarly for the D1, I1 and H1 scenario, the trajectories follow the main pattern of the Rim Current (Fig. 3c). Particles are flowing preferably in the west gyre without showing the details of the mesoscale structures. Each 200th particle trajectory from the beginning of May to the end July for all released particles since the start of the simulation is presented in the lower panel of Fig. 6 (35 in total per each sub-plot). The trajectories are also tend to follow the Rim Current pathway (Fig. 3d). However, the effect of mesoscale cyclonic eddies on the particle trajectory in the easternmost part of the basin is well visible for all scenarios.

**Fig. 7:**
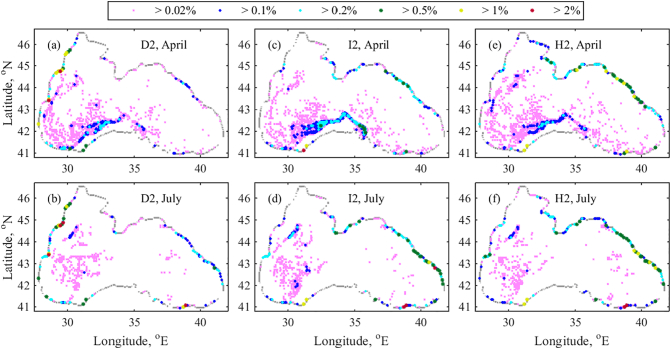
RPD (%) for 2018 in the case of beaching particle behaviour near the coast. (a) D2 at the end of April and (b) – end of July; (c) and (d) – I2; (e) and (f) – H2. RPD (%) bins are presented by different colours. For better plot readability, symbols representing higher RPD values have bigger size. (For interpretation of the references to colour in this figure legend, the reader is referred to the web version of this article.)

For the first three scenarios with beaching particle behaviour (upper panel in Fig. 7), we can emphasise the greater accumulation of particles in the south central part of the Rim Current in April, as in the case of bouncing particles. Moreover in July a large particle accumulation is forecasted in the same location along the NWS as for the bouncing particles. It appears that these two debris locations are independent of the coastal particle behaviour. A possible explanation for the debris formation between 30 and 31°E and 44 and 45°N in July includes the presence of the energetic Sevastopol anticyclonic eddy (Fig. 3d). With it, the particles are transferred from the eastern part of the shelf and shelf break to the western part of the Rim Current. At the same time, there are fewer particles in the rest of the inner basin due to high particle beaching rate.

The preferred areas for coastal beaching, however, depend strongly on the location of the particle release zones. For D2 in April, the zones with high number of beached particles are located north and south of the Danube, as well as in several places on the western and south-western coast. Particles are stacked south and north of the Danube, since the Danube plume can move in both directions depending on prevailing wind conditions. The plume is usually directed to the south, along the western coast. As we have already explained in Section 2.1, all particles reaching the beach are counted by the end of the year. In July, further particles are beached south of the Danube. Then, on the south-eastern beaches (~ 39°E) and at east and north-eastern coast, due to favourable mesoscale cyclonic circulation in the region (Fig. 4b).

Considering the I2 in April, it is found that more particles are accumulated in the south central Rim Current area and on the north-eastern coast than in the D2 (Fig. 7a and c). Fewer beached particles appear on the western and north-western coasts. Similar to the D2 and H2, debris in the western gyre is destroyed due to the Rim Current disintegration in July (Fig. 3d). In addition, a large quantity of released particles are transported to the eastern coast. Particles from H2 behave just as in I2, with the only difference that fewer particles are accumulated along the south central Rim Current (similar to the D2) and more on the north and north-eastern coast at the end of April. It is worth to note that, most particles are expected to reach the north and north-eastern coast for H2 (Fig. 7e). Further until the end of July, the eastern coast appears to be the particle preferred zone for all release scenarios (lower panel in Fig. 7). In summary, the main difference between all beaching scenarios is the high level of beached particles on the western coast in D2.

Regarding the mean stranding time in 2018, our model gives about 198 (±2) days for D2, 209 (±2) days for I2, and 200 (±2) days for H2. The above values are one order of magnitude greater than the stranding time in Stanev and Ricker (2019). In addition, our results for marine litter accumulation zones in the Black Sea completely disagree with Stanev and Ricker (2019) conclusions. They stated that - no pronounced floating marine litter accumulation zones and horizontal gradients were found; and floating marine litter concentrations decreased in the eastern and northern areas and increased along the western coasts. The reasons for the lack of accumulation zones and differently simulated litter distribution through the basin can be attributed to the circulation model results (http://marine.copernicus.eu/) linked with the Lagrangian model. The surface currents used by Stanev and Ricker (2019) seem to not properly represent the system circulation and in particular, the Rim Current speed and pathway. At the same time, on the base of a global circulation model, Lebreton et al. (2012) clearly indicated the existence of litter accumulation zones in the Black Sea. The resultant concentration of particles after 30 years of release and circulation for the two land based release scenarios (Danube and Istanbul) show accumulation zones in the western gyre and in the easternmost part of the basin. Extremely high particle concentrations are expected in several locations in the south-western and south-eastern coasts (It is better seen on the European regional map included in the Supplementary data).

### 5.2 Frequent debris locations

Annual bouncing scenarios are repeated for 20 years (1999–2018). The particle distribution is recorded on July 31st and December 31st for all three release scenarios. RPD time series from different bouncing scenarios do not show significant differences (significance level of 0.05). For this reason, we analyse results from the D1 only, since it is the top release point into the Black Sea (Halpern et al., 2008).

**Fig. 8:**
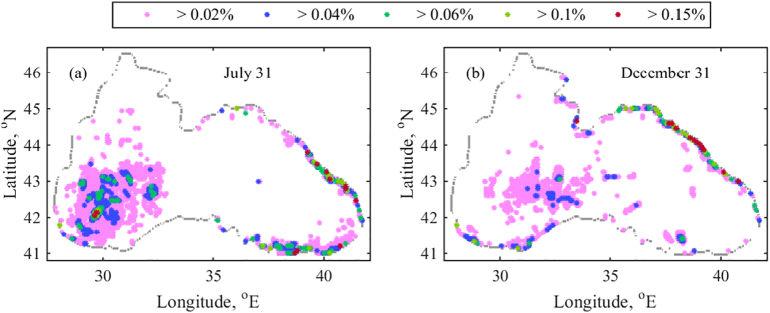
Climatological mean RPD (%) over 1999–2018 for D1. (a) July 31st and (b) December 31st.

The climatological RPD (%) averaged over 20-year period (Fig. 8) demonstrates a high concentration of accumulating particles in the inner western basin. Particularly, the retention zone at the end of July covers the area approximately located between 29 - 33°E and 41.2–43.5°N (Fig. 8a). In December the retention zone is shifted to the east covering a smaller area ~ 29.5–34°E and 42–43.5°N with lower particle concentration (Fig. 8b). The location and coverage of retention zones are easily attributable to the location and coverage of the western gyre in July (e.g. as in Fig. 4b). The RPD of the western retention zone is lower in December because of the stronger winter mixing. Convective mixing in winter, due to surface water cooling, causes an up/downwelling amplification in the entire basin. Thus, the debris zones in the basin interior are destroyed. Another evident accumulation zone in the western basin is located onshore in the vicinity of Bosphorus Strait. Particle retention is expected almost always in this area, as both records (late July and December) indicate a high concentration of particles. The Rim Current pathway is the main factor affecting particle retention. Particles move south along the western coast by the Rim Current. Approaching the Bosphorus, most of them are sharply turned east. However, another part is moving back to the NWS along the western coast by the help of Bosphorus eddy (Fig. 3d). The retention zone is formed between the location, where the Rim Current changes its direction toward east and the Bosphorus anticyclonic eddy. Owing to the Bosphorus eddy, the western coastal and shelf areas from ~42.3°N upwards are clear of marine debris almost always.

Looking at particle distribution in the eastern basin, it is evident that a large retention zone is shaped along the south-eastern and eastern coast in July. The easternmost basin is usually occupied by energetic mesoscale cyclonic eddies, formed between the coast and Eastern gyre in summer and autumn (Fig. 3b and d). As their centres move in a counter clockwise direction, they transport the particles incoming from the Rim Current to the shore rather than collect them. In this way, these mesoscale cyclonic eddies support the debris formation along the coast. In winter and spring, on the contrary, the integrated and strong Rim Current or two gyre system inhibit mesoscale circulation pattern formation and wash out marine debris from the south-eastern zones. Our results are in agreement with the observed decrease of microplastic concentration onshore and offshore along 30–36°E in February when compared to concentrations in the beginning of November (Aytan et al., 2016). Microplastics were assessed from zooplankton samples taken during two cruises along the south eastern coast of the Black Sea (see in Fig. 1 for the location of survey points). Average microplastic concentration in November (1.2 ± 1.1 × 10^3^ item m^−3^) was twice higher than in February. Typically in November, the mesoscale cyclonic motion in the easternmost part of the basin is still active (Fig. 3b), so floating debris is likely to accumulate along the south-eastern coast.

Along the eastern and north-eastern coast debris formation is expected in all seasons. Moreover, several debris zones with RPD > 0.15% can be seen in Fig. 8a, which move north at late December (Fig. 8b). According to the data from aerial surveys for spring-autumn 2004–2005 presented in BSC (2007), the maximum number of marine litter particles occurred in the southern part of the Russian Black Sea near Sochi (39.72°E and 43.59° N) and between Sochi and Tuapse (39.08°E and 44.1°N) (see in Fig. 1 for the location of survey points). The number of marine litter sightings decreased in the northern Black Sea areas and up to the southern boundary of the Kerch Strait near Cape Panagia (36.65°E and 45.18°N). The above observations support our suggestion that in the summer there is a higher particle density along the eastern coast between 42 and 43.8°N, than between 43.8 and 45°N (Fig. 8a). The Rim Current is powerful in December and the strong northerly and north-easterly winds that prevail in winter are blowing in the opposite or lateral direction of the Rim Current path. The combination of the Rim Current strength and winter wind causes the particles to move north and to form accumulations along the north and north-eastern coast.

**Fig. 9:**
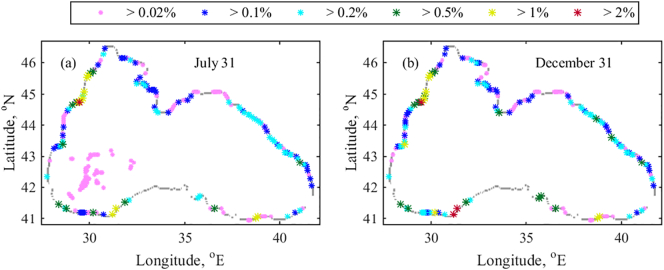
Climatological mean RPD (%) over 1999–2018 for D2. (a) July 31st and (b) December 31st. Note, that symbols representing higher RPD values have bigger size.

Climatological mean RPD (%) over 1999–2018 for D2 is shown in Fig. 9. It is evident that the major accumulation zones (RPD > 1%) are in the vicinity of the Danube, Cape Kaliakra (28.47°E, 43.37°N), Ereğli (31.3°E, 41.2°N), Zonguldak (31.5°E, 41.3° N) and Tirebolu (38.87°E, 41.07°N). While the influence of the release location (Fig. 2a) for the nearby beaching particles is obvious, the hot spot beaching sites at the Anatolian coast are independent of the release location (lower panel in Fig. 7). RPD in Ereğli and Zonguldak is between 1 and 2% at the end of July and >2% in December due to further particle accumulation over the year. Interestingly, both regions are considered solid wastes disposal sites with uncontrolled disposal (BSC, 2007). Information about the origin and type of disposal is not published. Yet, our results indicate that the floating litter tends to store in these locations. Regarding the location around Tirebolu, where our findings suggest the accumulation of debris, Aytan et al. (2016) observed a high microplastic concentration at this site in both surveys (November 2014 and February 2015).

A minor debris accumulation zone (RPD is <0.1%) is forecasted to form in the centre of the western gyre in July, which vanishes in December. Flowing along the western coast with the NWS fresh water plume, the particles released from the Danube source (Fig. 2a) tend to beach by 43.2°N. Then, along the Bulgarian and Turkish coast (by ~ 41.6°N) there are hardly any beached particles. Our results are in accordance with the published observations (Simeonova et al., 2017) that foreign based litter is not found on Bulgarian beaches and coastal areas (Fig. 1). Along the south-western Turkish coast, an intermediate coastal beaching intensity is suggested. In this area, the Rim Current changes sharply its direction and carries particles partly on land. It is worth noting that the same zone of the south-western coast is susceptible to generation of retention zones in the case of particle bouncing (Fig. 8). Our simulations are supported by the field data estimated from surveys (Topçu et al., 2013) on 10 beaches of the Turkish Western Black Sea coast (see in Fig. 1 for the location of survey points). It was found that about a half of identifiable labelled litter is foreign. At about 32–35.5°E, the Anatolian coast is clear of particles (RPD <0.02%), and otherwise only a few locations are favoured for litter beaching (RPD ~0.5%).

**Fig. 10:**
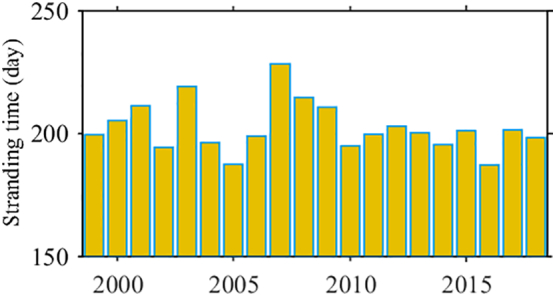
Annual mean stranding time from D2.

Particle beaching along the entire eastern coast is well captured in Fig. 9. RPD of beached particles is already between 0.2 and 1% at late July, which is followed by a small increase in a few sites by the end of December. High concentrations of beached particles along the eastern and north-eastern coast are expected, since even particles with a bouncing coastal behaviour tend to accumulate there. Along the northern coast, fewer particles are beached west and east of the Kerch Strait and more at the Crimea Peninsula. Note that the amount of beached particles at the NWS coast of the Crimea Peninsula at the end of December is almost the same as in July. Only in a site south of Sevastopol RPD increases twice from July to December. Stranding time for D2 seems to vary weakly over 1999–2018 period (Fig. 10), with an average of about 202 days. Not surprisingly, there is a negative correlation between the stranding time and Danube discharge over this period (Pearson correlation coefficient is equal to −0.6 and *p-value* = 0.006). No significant trend of the stranding time is identified.

### 5.3 Trends

**Fig. 11:**
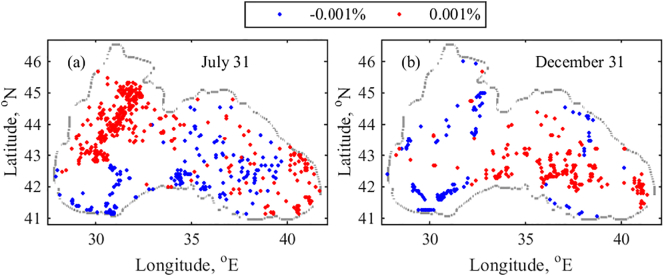
Trends of RPD (% per year) with p-value <0.05 over 1979–2018 for D1. (a) July 31st and (b) December 31st. The trends are grouped in 2 bins: −0.001–0% (blue) and 0–0.001% (red). (For interpretation of the references to colour in this figure legend, the reader is referred to the web version of this article.)

To study the changes in floating litter accumulation over a long term period, we repeat D1 annual simulations from 1979 to 2018. Linear trends of 40-year RPD time series are then computed on July 31st and December 31 for each grid box, and RPD percentage change per year with p-value <0.05 is plotted (Fig. 11). The trends are grouped in 2 bins: −0.001–0% (negative) and 0–0.001% (positive), where the blue colour represents the negative values and red – positive values for both plots in order to make more apparent the comparisons between RPD trends in particular months. For several grid boxes, which are spread over the entire basin, low increasing/decreasing trends are established. Larger patches of boxes with increasing trends are identified on the NWS and shelf break in late July. The increasing RPD trend could be a result of the NWS plume pathway changes. The Danube plume moves toward the north and north-eastern part of the NWS more frequently than directly to the south. The river waters remain locked in the north and north-eastern part of the NWS up to a month or longer, where the river-borne litter is horizontally and vertically mixed. In several locations of the preferred accumulation zones along the easternmost basin (Fig. 8a), an increasing trend is found (Fig. 11a). In December, patches of zones with an increasing trend can be seen in the eastern basin and with a decreasing in the western basin (Fig. 11b). It appears that particles tend to accumulate more in the eastern inner basin.

## 6 Conclusions

Distribution of marine litter in the Black Sea, studied here, is based on the assumption that floating litter particles are transported mainly by surface currents. We do not consider Stokes drift, wind drag, biofouling and sinking. Our results also closely resemble occasional field data (Topçu et al., 2013; Aytan et al., 2016; Suaria et al., 2015; BSC, 2007), particularly in terms of the accumulation zone locations. Six different scenario are considered to study the influence of litter input location and coastal particle dynamics (bouncing or beaching) on the litter spreading and accumulation. In general, the dispersion of litter is almost entirely independent of the location of litter input to the sea. Within a few months from the start of the simulation period, the floating litter displays similar patterns independently of the litter source. The mean particle stranding time is also almost independent of the litter source (~ 200 days). The beaching coastal behaviour decreases the litter density in the basin due to litter accumulation on the beach, however the litter distributions in the basin remain similar as in the case studying bouncing coastal behaviour. We can recognise a big retention zone in the centre of the western gyre in summer, which is shifted eastwards in winter. A stable retention zone along the south-western coast is simulated for both coastal particle behaviour in all seasons. Most of the retention zones along the south-eastern coast, existing in summer, are destroyed in winter and the litter is moved northwards along the eastern coast. Several hotspots of litter accumulation are identified, namely the south-western Turkish coast (area between the Bosphorus Strait and Sakarya river) and the eastern coast, which retain litter continuously. The most significant yearly increasing trend of the litter accumulation is identified in the NWS and shelf break zone in late July. Increasing trends in accumulation in the eastern basin cover smaller areas. Our model qualitatively describes the spreading and accumulation of floating debris and can provide a reasonable assessment on the relative impacts of regional inputs to the Black Sea pollution However, there is a need for a more accurate model that can account for a wider range of processes affecting the spread of marine litter.

Hindcasting performed herein is particularly useful for identification of accumulation regions. The same approach can further be used to identify where accumulation zones are most likely to occur. Future scenario simulations, coupled with various management options (e.g., related to litter management or plastic usage), can provide valuable stakeholder information aimed at maintaining a healthy environmental status of European waters. Additionally, they can contribute to the development of concepts for future research within the scope of Marine Strategy Framework Directive (MSFD, 2008/56/EC).

## CRediT authorship contribution statement

**S. Miladinova:**Conceptualization, Methodology, Software, Writing - original draft.**D. Macias:**Conceptualization, Methodology, Writing - review & editing.**A. Stips:**Supervision, Project administration, Funding acquisition, Writing - review & editing.**E. Garcia-Gorriz:**Data curation, Visualization, Software.

## Declaration of competing interest

The authors declare that they have no known competing financial interests or personal relationships that could have appeared to influence the work reported in this paper.
